# Humic Acid Enhances Wound Healing in the Rat Palate

**DOI:** 10.1155/2018/1783513

**Published:** 2018-08-01

**Authors:** Metin Çalışır, Aysun Akpınar, Ahmet Cemil Talmaç, Aysan Lektemur Alpan, Ömer Fahrettin Göze

**Affiliations:** ^1^Department of Periodontology, Faculty of Dentistry, Adiyaman University, Adiyaman, Turkey; ^2^Department of Periodontology, Faculty of Dentistry, Cumhuriyet University, Sivas, Turkey; ^3^Department of Periodontology, Faculty of Dentistry, Yuzuncu Yil University, Van, Turkey; ^4^Department of Periodontology, Faculty of Dentistry, Pamukkale University, Denizli, Turkey; ^5^Department of Pathology, Faculty of Medicine, Cumhuriyet University, Sivas, Turkey

## Abstract

**Introduction:**

Humic acid was previously shown to enhance cutaneous wound healing and show antibacterial properties; however, it has not been used for wound healing in the oral cavity. Thus, the goal of this study was the evaluation of the effect of the humic acid on the healing of excisional wounds in an experimental rat study.

**Materials and Methods:**

A circular wound on mid-palatal surfaces was made on a total of 77 Wistar rats by using a 3-mm biopsy punch under anesthesia. The animals were divided into 4 groups as baseline, saline control (0.09%), chlorhexidine gluconate (0.05%), and humic acid (80 mg/kg) and were treated with these materials for 7 days.

**Results:**

The rats were observed for 3 weeks in order to track the wound closure rates. Both humic acid treatment and chlorhexidine gluconate treatment resulted in statistically significant enhanced rate of wound closure compared to the saline control on both the 1st and 2nd weeks of treatment. Humic acid treatment for the wounds in the palate resulted in enhanced recovery compared to not only saline control but also chlorhexidine gluconate treatment.

**Conclusion:**

In this study, humic acid was shown to enhance healing of oral wounds for the first time in the literature. These findings indicate that humic acid can be used as an alternative to current treatment methods for oral wounds.

## 1. Introduction

Wounds in the oral cavity constitute an important health concern for many people due to the warm oral microenvironment that is home to many bacteria and the constant wear and tear that occurs due to physical activity caused by eating and drinking [1, 2]. Wound healing is a complex process that is characterized by three stages as inflammation, proliferation, and remodeling [3]. During the inflammation phase, the wound area is protected against pathogens and the dead cells are removed, whereas, during proliferation phase, the cells that secrete extracellular matrix materials proliferate and secrete high amounts of fibrous extracellular matrix proteins in order to rapidly block and protect the wound area against pathogens [4]. During remodeling phase, these fibrous blocking components are removed and more functional tissue is generated.

Since the highly humid and warm environment of the oral cavity is a supportive environment for microbial growth, it is customary to use antibacterial products to aid in rapid and infection-free wound healing for wounds in the palate [5, 6]. Other than the antibacterial products that are already commercially used, few factors were previously shown to aid palatal wound healing [5–7].

Humic substances, which are present mostly in lignite, peat, soil, and water, have antiviral, antibacterial, antitoxic, antiulcerogenic, antiarthritic, antiallergic, immunomodulatory, and anti-inflammatory properties [8–15]. Microorganisms convert plant and animal tissue into peat [16], and humic substances such as humus, peat, sapropel, and mumie have been used in medicine for different applications against different illnesses as far as 3000 years ago [11]. The toxicity of humic acid is very low [17]. Use of humate results in reduction in paw volume of the carrageenan-induced edema in rats [18] and sodium humate was shown to enhance wound healing in rats [19].

In the oral cavity, we have previously shown that humic acid prevents alveolar bone loss and reduce inflammation in rats [20]. In addition, carbohydrate-derived fulvic acid, which is a major constituent of humic acids, has been shown to have a broad-spectrum antimicrobial activity against orally active microorganisms [21]. Although these results suggest the possibility that these specific properties of humates may be useful in wound healing in the palate, currently, there is no evidence showing the effects of humates on wound healing in the oral cavity. Thus, the aim of this study was the evaluation of the effect of the humic acid on the healing of excisional wounds in the palate of rats.

## 2. Materials and Methods

### 2.1. Animals and Study Groups

The study protocol and experimental design were approved by the Animal Ethics Committee of Cumhuriyet University School of Medicine (approval number: B.30.2.CUM.0.01.00.00-50/59, 312). In total, 77 three-month-old male Wistar rats were used in the experiment. Their body weight ranged from 280 to 320 g at the beginning of the experiment. Rats in each group were fed in different cages under the same conditions in a well-lit and well-ventilated room. All rats were fed* ad libitum* and kept at 12 h/12 h light/dark cycle and at 21 ± 1°C temperature and 40–60% humidity. Rats were acclimated to their living environment for 10 days prior to the study to alleviate stress related interference with experimental set-up. The experimental stages of this study were performed in the Animal Laboratory of Cumhuriyet University's Faculty of Medicine. The animals were randomly divided into four groups as follows:

- Control (C) group (n = 5)
- Saline (0.9%) (S) group (n = 24)
- Chlorhexidine gluconate (0.05%) (CHG) group (n = 24)
- Humic acid (HA) group (n = 24)

Each main group was divided to three subgroups containing 8 rats in each to observe changes after 1st, 2nd, and 3rd weeks.

### 2.2. Formation of Experimental Palatal Wound Surface

After an adaptation period of 10 days, animals were anesthetized with xylazine hydrochloride (Rompun; 10 mg/kg, Bayer Animal Health GmbH, Leverkusen, Germany) and ketamine hydrochloride (Ketalar; 40 mg/kg, Eczacibasi Ilac Sanayi, Istanbul, Turkey) intraperitoneally. A punch biopsy tool with 3 mm diameter was used to create a standardized circular wound outline on the anterior palate in the mucoperiosteum of midline of the hard palate. The soft tissue was removed by sharp dissection to expose the underlying bone. Cotton gauze was placed over the wound until hemostasis was achieved. No medication was used throughout the experiment.

### 2.3. Preparation of Humic Acid

Humic acid was obtained from peat coming from the western Black Sea region and was diluted in sterile saline solution to reach the study concentrations (80 mg/kg) [20]. The concentration of the trace elements in the humic acid solution such as Si, Se, Ca, Mg, Fe, and Zn is provided in Table 1. Titrimetric method was used for the assessment of Cl, whereas inductively coupled plasma-atomic emission spectrometry (ICP-AES) was used for all the other elements.

### 2.4. Study Process

Five animals were sacrificed immediately to get the baseline values (C group). The remaining 72 animals were randomly divided into three experimental groups. 0.5 mL of 0.09% saline solution, 0.05% chlorhexidine gluconate (Irrisept, Irrimax Corporation, Innovation Technologies, Inc., Lawrenceville, GA), or 80 mg/kg humic acid preparation was applied to the respective wound site once daily for 1 min each day by using cotton pellets. Eight animals from each group were sacrificed at 7, 14, and 21 days postoperatively. The maxillae were dissected out and the samples were photographically assessed and compared with the histological findings.

### 2.5. Photographic Assessment

Photographs of the specimens were taken (25X magnification) using a stereomicroscope (Stemi DV4, Carl Zeiss, Jena, Germany). The surface area of the wound was morphometrically measured using the "Biowizard - D Winter, Version 3" software. The photographic assessment was performed by a single examiner (Dr. Talmac) who was unaware of the identity of samples.

### 2.6. Histopathological Assessment

Histological analysis was performed by a single examiner (Dr. Goze) who was also blinded to the identity of samples. Specimens were fixed in 10% neutral formalin for 48 h. The samples were then decalcified in formic acid (10%) and nitric acid (10%) for 72 h. Then, the samples were embedded in paraffin. 5 *μ*m serial sections were prepared perpendicular to the palatal midline at the greatest diameter of the wound by using a microtome. The sections were stained with eosin and hematoxylin. Slides were evaluated for histological changes under light microscopy (Nikon Eclipse, E 600, Tokyo, Japan).

### 2.7. Statistical Analysis

The control and experimental group data were compared with each other and to the baseline values. Statistical analysis was done using the SPSS software and the GraphPad Prism program. Two-way ANOVA or one-way ANOVA with Tukey's multiple comparisons analysis and Student's t-test were applied.

## 3. Results

**Figure 1:**
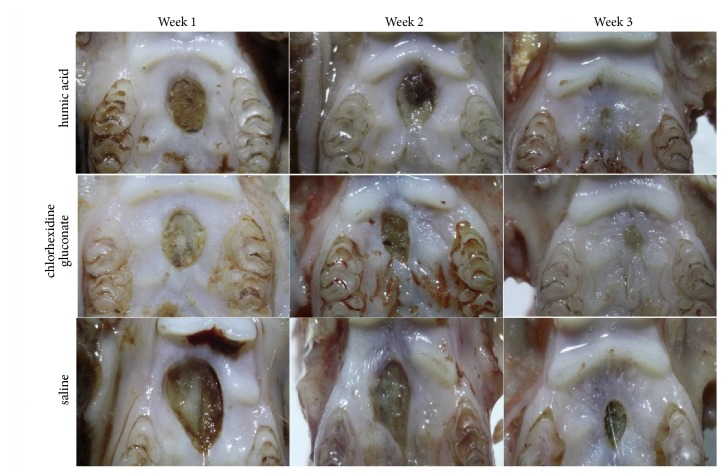
The representative light microscopic photographs of the wound areas on weeks 1, 2, and 3.

The humic acid was obtained as a paste and was further diluted at a concentration of 80 mg/kg in saline solution. After surgical operation, photographs of the wound areas were obtained with a light microscope and the images were measured by an observer who was blind to the study groups. The negative control “saline solution”, the positive control “chlorhexidine gluconate solution”, and the humic acid solution were applied to the wound area daily for one minute. The representative pictures of the wound areas that were taken with a light microscope are shown in Figure 1.

After the first week of treatment, there was a statistically significant difference between groups by one-way ANOVA analysis with a p value of 1.89 x 10^−17^ (Table 2). When the groups were individually compared with each other through Tukey's multiple comparisons test, there was a statistically significant difference between the saline control and the humic acid group as well as saline control and chlorhexidine gluconate group, with p values 2.06 x 10^−13^ and 5.01 x 10^−13^, respectively. On the other hand, there was no statistically significant difference between the humic acid group and the chlorhexidine gluconate group after one week of treatment.

At the end of the second week of treatment, the wound areas were measured again, and the groups were statistically analyzed with one-way ANOVA. There was a statistically significant difference between groups with a p value of 4.61 x 10^−10^ (Table 2). Similar to the first week of treatment, there was a statistically significant difference between the saline control and the humic acid group as well as the saline control and the chlorhexidine gluconate group, with p values 9.21 x 10^−8^ and 3.05 x 10^−8^, respectively. However, at the end of the two weeks of treatment, there was still no statistically significant difference between the humic acid group and the chlorhexidine gluconate group.

The study was performed for three weeks, and at the end of the study, the wound area measurements were again compared with one-way ANOVA and multiple comparisons between groups were done with Tukey's test. Overall, there was a statistically significant difference between groups with a p value of 1.94 x 10^−14^ (Table 2). Similar to first and second week measurements, there was a statistically significant difference between the saline control and the humic acid group as well as saline control and chlorhexidine gluconate group. The p values of these comparisons were 3.83 x 10^−11^ and 3.78 x 10^−10^, respectively. Strikingly, at the end of three weeks, there was also a statistically significant difference between the humic acid group and the chlorhexidine gluconate group with a p value of 0.0001 (3.95 x 10^−5^) as well.

When all three weeks' measurements were compared by using two-way ANOVA with repeated measures, there was a statistically significant difference between not only weeks but also treatment groups, with p values of 1.55 x 10^−41^ and 1.68 x 10^−64^, respectively.

**Figure 2:**
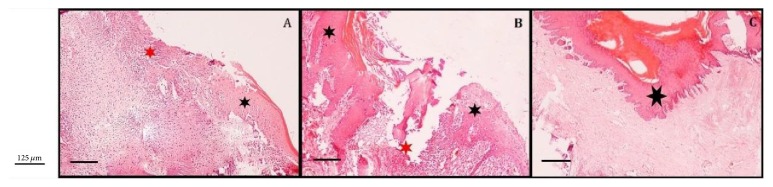
The representative histological image of the wound areas. (A) Representative histological image of the humic acid treated wounds at 1st week. The red star shows the wide necrotic and severely inflamed area, while the black star points to the mucosal epithelium. (B) Representative histological image of the humic acid treated wounds at 3rd week. The red star shows the mildly inflammatory area and granulation tissue, while the black stars show the constricted mucosal epithelial layer. (C) A representative section of humic acid treated tissues at the end of the study. Black star shows complete mucosal epithelial repair and healing.

When the tissue sections were analyzed after being processed with hematoxylin and eosin staining, the sections from the one-week treatment group showed necrotic and inflamed areas. After three weeks of treatment with humic acid, however, the inflammation areas were much reduced and granulation tissue with constricted mucosal epithelial layer was observed. At the end of the study, sections from the humic acid treated animals showed complete mucosal epithelial repair and healing (Figure 2).

## 4. Discussion

In this study, humic acid was shown to enhance healing of oral wounds for the first time in the literature. The experiments were performed in rats and humic acid treatment was compared to saline treatment and traditional chlorhexidine gluconate treatment.

Bacterial microflora in the oral cavity is very diverse and these bacteria colonize the wounds [22]. Wounds in palate are usually treated with antibacterial treatments to prevent infections. In this study, we also used an antibacterial material, chlorhexidine gluconate, as a positive control. Humic acid was also previously reported to have antibacterial properties [9, 12]; therefore, this control enabled us to compare the effectiveness of the antibacterial treatment for wound healing. In the first two weeks of treatment, we observed the positive effects of the antibacterial on oral wound healing for both humic acid treatment and chlorhexidine gluconate treatment, which were much more effective in enhancing wound healing than the saline control. On the other hand, within the first two weeks, there was almost no difference between the humic acid treatment group and chlorhexidine gluconate treatment group, which shows the importance of antibacterial properties for the initial stages of wound healing. The histological analysis which showed granulation tissue with constricted mucosal epithelial layer and complete mucosal epithelial repair and healing after humic acid treatment also support the critical effect of humic acid in wound healing in palatal wounds.

Humic acid was also shown to have anti-inflammatory properties, which can aid in healing of the wounds on different tissues [10, 15, 18, 23]. This property might be more influential at later stages of wound healing, since we observed that the humic acid treated wound healed statistically significantly faster than the chlorhexidine gluconate treated wounds on the third week of the treatment. In addition, at the end of three weeks of treatment, the humic acid treated wounds showed reduced inflammation areas compared to both the saline control and the chlorhexidine treated group when the samples were analyzed through histological staining.

Humic acids can have a positive effect on wound healing and cancer therapy, as suggested by Jurcsik [24]. The healing process requires extra oxygen, and this demand appears in the first minute after wounding due to phagocytosis, the main event in wound healing process, which is very oxygen-consumptive [24].

The composition of humic acid is complex and the samples from different areas are different from each other. Although drug substances that are prepared by using natural materials as starting materials are routinely used and are allowed to differ to a certain extent as batch-to-batch variations, it might be beneficial to use synthetic humic acid preparations for future wound healing experiments as alternatives to these natural samples to achieve more chemically defined drug products.

## 5. Conclusion

Overall, the results of this study showed that humic acid, which has previously been shown to have antibacterial and anti-inflammatory properties, enhances wound healing in the oral cavity. The humic acid treatment was even superior to chlorhexidine gluconate, which is widely used for the treatment of oral wounds. To the best of our knowledge, this is the first study to show that humic acid treatment can be used for the treatment of wounds in the oral cavity.

## Figures and Tables

**Table 1:** Composition of the humic acid sample.

**Analysis Parameters**	**Unit**	**Methods**	**Analysis Results W/W'**
Total humic acid	%	TS 5869 ISO 5073	6.5
Total P	%	ICP-AES	0.015
Total Si	%	ICP-AES	1.36
Total Se	mg/kg	ICP-AES	0.15
Total Ca	%	ICP-AES	1.26
Total Mg	%	ICP-AES	0.014
Total Fe	%	ICP-AES	0.056
Total Mo	mg/kg	ICP-AES	89.50
Total Zn	mg/kg	ICP-AES	31.13
Total Na	%	ICP-AES	1.79
Total Cl	%	Titrimetric	-

**Table 2:** Analyses of wound areas during 21 days of observation.

	**1st week of treatment**	**2nd week of treatment**	**3rd week of treatment**
**Control (mean ± sem)**	8.22	5.131	2.194
**Chlorhexidine gluconate (mean ± sem)**	5.276	4.02	1.215
**Humic acid (mean ± sem)**	5.193	3.97	0.7788
**F-value**	400.6	70.85	201.9
***R*** ^***2***^ **-value**	0.9745	0.8709	0.9506
**95**%** CI between control and humic acid**	2.637 - 3.251	0.8832 - 1.439	1.233 - 1.597
**95**%** CI between control and chlorhexidine gluconate**	2.720 - 3.335	0.8332 - 1.389	0.7970 - 1.161
**95**%** CI between chlorhexidine gluconate and humic acid**	-0.2234 - 0.3909	-0.2280 - 0.3280	0.2545 - 0.6180

sem = standard error of mean and CI = confidence interval.

## Data Availability

No data were used to support this study.
